# Effects of the Mediterranean Diet on the Components of Metabolic Syndrome Concerning the Cardiometabolic Risk

**DOI:** 10.3390/nu17020358

**Published:** 2025-01-20

**Authors:** Stefania Scaglione, Tiziana Di Chiara, Mario Daidone, Antonino Tuttolomondo

**Affiliations:** Internal Medicine and Stroke Care Ward, Department of Promoting Health, Maternal-Infant, Excellence and Internal and Specialized Medicine (Promise) G. D’Alessandro, University of Palermo, 90127 Palermo, Italy; stefania.scaglione@you.unipa.it (S.S.); tiziana.dichiara@unipa.it (T.D.C.); mario.daidone@unipa.com (M.D.)

**Keywords:** Mediterranean diet, metabolic syndrome, cardiovascular risk, type 2 diabetes, arterial hypertension, dyslipidemia

## Abstract

Metabolic syndrome is a cluster of risk factors, including abdominal obesity, insulin resistance, hypertension, dyslipidemia (intended as an increase in triglyceride levels and a reduction in HDL cholesterol levels), and elevated fasting glucose, that increase the risk of cardiovascular disease and type 2 diabetes. With the rising prevalence of metabolic syndrome, effective dietary interventions are essential in reducing these health risks. The Mediterranean diet, rich in fruits, vegetables, whole grains, legumes, nuts, and olive oil and moderate in fish and poultry, has shown promise in addressing metabolic syndrome and its associated components. This diet’s anti-inflammatory and antioxidant properties, primarily due to its unsaturated fats, polyphenols, and fiber, have improved blood pressure, lipid levels, and insulin sensitivity. Adherence to the Mediterranean diet has been linked to reductions in central obesity and insulin resistance, both key elements in managing metabolic syndrome. Regarding lipid management, the Mediterranean diet lowers triglyceride levels and low-density lipoprotein (LDL) cholesterol while raising high-density lipoprotein (HDL) cholesterol, enhancing lipid profiles. It also helps regulate blood glucose levels, reducing the likelihood of developing type 2 diabetes. Additionally, the diet promotes weight loss and improves body composition, particularly by decreasing visceral fat, a primary driver of metabolic syndrome according to IDF classification. The Mediterranean diet offers a holistic approach to managing metabolic syndrome and reducing the risk of related chronic diseases. Its positive impact on metabolic health, combined with lifestyle changes like increased physical activity, provides a sustainable method for addressing the global burden of this syndrome. This review aimed to summarize the positive effects of the Mediterranean diet on the component of the metabolic syndrome with subsequent positive effects on cardiometabolic risk profile.

## 1. Introduction

**Figure 1:**
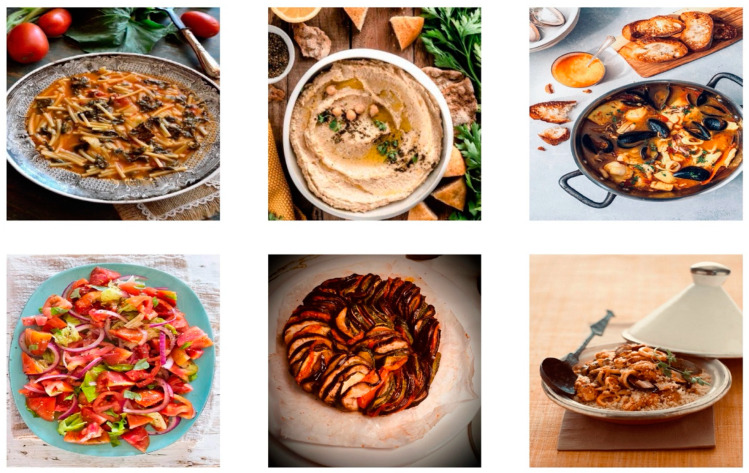
Typical dishes of Mediterranean diet.

The Mediterranean diet (Me-Di) has been widely recognized in numerous studies as a model for healthy eating. It is characterized by a high consumption of fruits, nuts, vegetables, legumes, cereals, fish, and other seafood, while the intake of dairy products, meat, and processed meats is kept low. The diet also includes moderate alcohol consumption, primarily in the form of wine during meals (see examples of typical Mediterranean diet dishes) (see Figure 1).

Research has shown that adherence to a Mediterranean-style diet can reduce the risk of cardiovascular disease, cancer, Alzheimer’s disease, Parkinson’s disease, and mortality related to cardiovascular diseases or cancer, as well as premature death in general. Additionally, further studies have indicated that greater adherence to this diet can help reduce the burden of carotid atherosclerotic plaque and provide protection against cerebrovascular major adverse cardiovascular events (MACEs) [1].

A recent randomized trial [2] aimed at evaluating the effectiveness of two Mediterranean diets for primary cardiovascular prevention found that patients adhering to the Mediterranean diet had a significantly lower risk of stroke. This result aligns with epidemiological studies that report an inverse relationship between adherence to the Mediterranean diet and stroke incidence [3].

Several studies have also highlighted the favorable metabolic outcomes associated with better adherence to the Mediterranean diet. It is therefore clear that following a Mediterranean diet has beneficial effects on metabolic syndrome, its individual components, and the associated cardiometabolic risk [4].

Given this evidence, the purpose of this narrative review is to summarize the available literature regarding the Mediterranean diet and metabolic syndrome, as well as the positive effects of adherence to the Mediterranean diet on the cardiometabolic risk profile and the metabolic components of the syndrome.

## 2. Mediterranean Diet from the Past to Now

Diet is from the Latin ’diaeta’, from the Greek ‘δίαιτα’ way of life. In ancient Greek medicine, the rules of life (diet, physical activity, rest) were designed to maintain health.

The Mediterranean diet represents the central historical and cultural heritage that tells the story of the peoples of the Mediterranean basin through the centuries.

More than a diet, the Mediterranean diet mirrors the eating and living style of Mediterranean countries with the use of olive oil in common. This diet is typical of those Mediterranean regions (Figure 1) where olive tree cultivation is widespread and olive oil is, therefore, the primary source of fats in the diet [5].

However, the Mediterranean diet variations in different Mediterranean countries, such as climate, geographic location, culture, socioeconomic factors, religious beliefs, and historical events, have influenced diet. For example, wine is crucial in northern Mediterranean countries (Spain, France, and Italy) but is absent in southern Mediterranean Arab countries for religious reasons. Meat consumption also varies: Arabs consume poultry and sheep, while European countries prefer red meat. Pasta is mainly present in Italy, while it is absent in Arab countries, where people prefer other cereals.

In the ’healthy Mediterranean diet’ [6], the most represented elements are cereals, legumes, fish, fresh fruit, nuts (especially walnuts because they are rich in omega-3 fatty acids), vegetables, wild plants, wine as an optional alcoholic beverage, and olive oil as the central element of the Mediterranean diet that unites all Mediterranean peoples (Table 1).

Since the second half of the twentieth century, there has been increasing interest in Mediterranean countries’ dietary habits and lifestyles. The ’seven country study’ (SCS) is the first to investigate diet, lifestyle, and other risk factors for cardiovascular disease in contrasting countries and cultures over time. In this study, it was observed that, in men aged 40 to 59 years in rural Mediterranean Europe (Dalmatia, Crevalcore, Montegiorgio, Crete, and Corfu), age-standardized mortality rates for all causes and coronary heart disease (CHD) were low compared to men in non-Mediterranean Europe (Eastern and Western Finland, Slavonia, etc.) [7].

Another study conducted in 1960 on dietary habits, understood as eating habits and lifestyle, observed an Italian Calabrian population in Nicotera. Nicotera was the third rural Italian area in the seven country study, which was examined in 1957 as a pilot study. The diet was predominantly composed of cereals, which provided 48% to 52% of the total energy intake, along with extra virgin olive oil contributing 14.5% to 16.6% of energy. Vegetables accounted for 5% to 7% of energy, while legumes supplied 4.4% to 6.6%. Moderate amounts of fruit (2.0% to 2.6% of energy), fish (1.6% to 2.0%), and red wine (4.2% to 6.0%) were also included. Conversely, the consumption of meat (2.6% to 4.0%), dairy products (1.3% to 1.8%), eggs (0.8% to 1.4%), and animal fats (1.0% to 2.0%) was limited. The men adhered to moderate physical activity, often engaging in labor-intensive tasks.

Meals were carefully prepared to include a complementary blend of nutrients, bioactive compounds, and other protective food elements, aided by the use of antioxidant-rich herbs and spices. Both the selection of ingredients and the proportions in traditional recipes played a significant role in ensuring balanced nutrition.

Examples of meal combinations included pasta with tomato sauce and vegetables or fish, as well as soups made with pasta or other grains, pulses, and vegetables, often enriched with onion and garlic. Whole-grain bread, made from stone-ground flour, was a staple and was consumed with a variety of foods, including fruit. This approach fulfilled nutritional needs while also ensuring sensory satisfaction.

In Nicotera’s pilot study, the prevalence of chronic degenerative diseases in men aged 45–64 was very low, hypertension, overweight, and obesity were rare, and cancer was virtually absent; only a few men were light smokers. Other population groups surveyed in Central and Southern Italy followed a diet similar to Nicotera’s. However, eating habits have changed drastically over the past four decades, and many beneficial nutritional characteristics have been abandoned [8].

During this study, an index system known as the ’Mediterranean Adequacy Index (MAI)’ was developed to establish how similar a diet is to the reference Mediterranean diet [9].

(% of energy in bread, cereals, legumes, dry and fresh

potatoes, vegetables, fresh fruit, nuts, fish,

wine, and vegetable oils)

MAI = —————————————————————

(% of energy in milk and dairy products, meat, eggs,

animal fats and margarines, sweet beverages,

cakes, pies, and cookie sugar)

**Figure 2:**
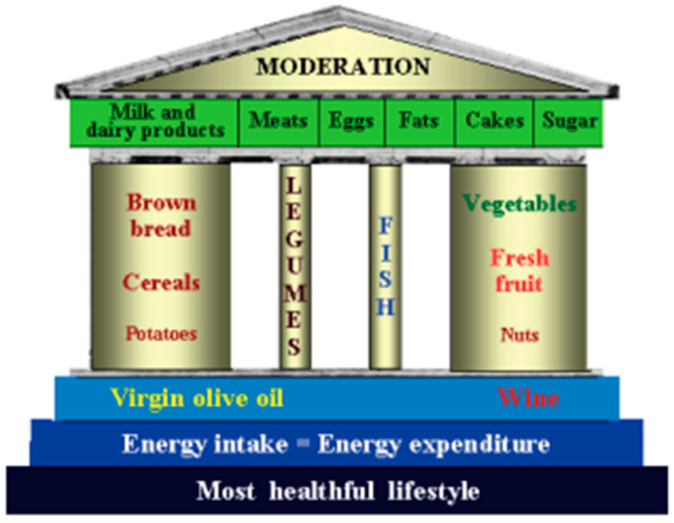
The Healthy Italian Mediterranean Diet Temple Food Guide [9].

The authors proposed a clear and straightforward food guide to preserve these eating habits even though globalization and increasing technologies are drastically changing ‘the Reference Italian Mediterranean Diet (RIMD)’. The idea of the Temple of the Healthy Italian Mediterranean Diet was inspired by Simopoulos’s article on the Mediterranean Food Guide [10]. Simopoulos proposed a food guide consisting of seven Greek columns (Figure 2). The main dishes are indicated for each column, corresponding to a week’s day. The visualization of this food guide in the form of a Greek column includes the concepts of genetic individuality, the principles of moderation, variety, proportionality, and balancing energy intake with energy expenditure. The temple is based on food, not food groups.

Over the years, several studies have confirmed what these pioneering studies had begun to investigate. However, in addition to the quality of food, which significantly influences the onset of chronic diseases such as diabetes, obesity, and increased cardiovascular risk, lifestyle also plays a crucial role. Today, mechanized farming systems, large industries, means of transport, and comforts have significantly reduced daily physical activity; there is an increasing tendency to do static work and to look for the lowest energy expenditure to carry out any activity; outdoor hobbies are less and less chosen compared to hobbies done at the computer or rather zapping on TV. The increase in sedentariness has done nothing but reduce the energy expenditure of individuals, and, as a result, we are seeing weight gain to the point of obesity.

## 3. Metabolic Syndrome and Cardiovascular Risk

### 3.1. History of the Relationship Between Metabolic Syndrome and Cardiovascular Risk

The first evidence found between abdominal adiposity, atherogenic and/or diabetogenic variables and/or obstructive sleep apnea is very ancient. Both Hippocrates, in the fifth century BC, the Greek-Egyptian philosopher Athenaeus of Naucratis, in the third century BC, and the Roman historian Polybius, in the second century BC, described familial obesity, with abdominal distribution, hyperphagia, joint tophi (indicative of hyperuricemia), and lethargy (i.e., obstructive sleep apnea) in the dynasty of the Ptolemaic pharaohs.

Two thousand years later, in 1765, Giovanni Battista Morgagni described two patients with abdominal obesity, including a 74-year-old woman who died of a stroke cerebri. The woman showed an android constitution with autopsy evidence of intra-abdominal fat accumulation, dilated cardiac hypertrophy, signs of hypertension and pulmonary edema, and carotid and vertebral atherosclerosis.

From the 20th century onwards, studies began defining the most frequent associations between diabetogenic and atherogenic variables and their relationship with obesity.

During the First World War, the Viennese internist Karl Hitzenberger observed that elderly diabetic subjects were hypertensive [11], a finding confirmed shortly afterwards by Spanish endocrinologist Gregorio Marañón [12] and by Swedish internist Eskil Kylin, who, in 1923, also noted the association with gout, defining a hypertension–hyperglycemia–hyperuricemia syndrome [13].

Then, in 1936, Britain’s Sir Harold Himsworth first demonstrated the presence of sensitivity or insensitivity in people with diabetes to exogenous insulin for glucose utilization, introducing the concept known today as insulin resistance [14].

Approximately ten years later, in 1947, French internist Jean Vague first defined the phenotypes of human obesity, pointing out that ‘android’ (as opposed to gynoid) adiposity, characterized by accumulation in the upper torso and, in particular, increased abdominal girth, was associated with atherosclerosis, diabetes, and gout, suggesting that visceral obesity predisposed to cardiovascular and glycemic disorders [15].

Starting in the mid-1960s, several dysmetabolic associations, all of which we now know converge in MetS, were described by various authors until, in 1988, Gerald M. Reaven brought together under the eponym ‘syndrome X’ both Himsworth’s concept of insulin resistance and hyperinsulinemia, dyslipidemia (hypertriglyceridemia VLDL and hypercholesterolemia HDL), and hypertension, resulting in a predisposition to atherosclerosis and coronary heart disease [16,17].

Finally, in 1989, Norman Kaplan formalized the requirement of splanchnic and subcutaneous abdominal adiposity (so-called central adiposity) for the diagnosis of MetS [13], and, in 1991, De Fronzo and Ferranini introduced the term ‘insulin resistance syndrome’ as a synonym for metabolic syndrome [18].

### 3.2. The Modern Definition of Metabolic Syndrome

**Figure 3:**
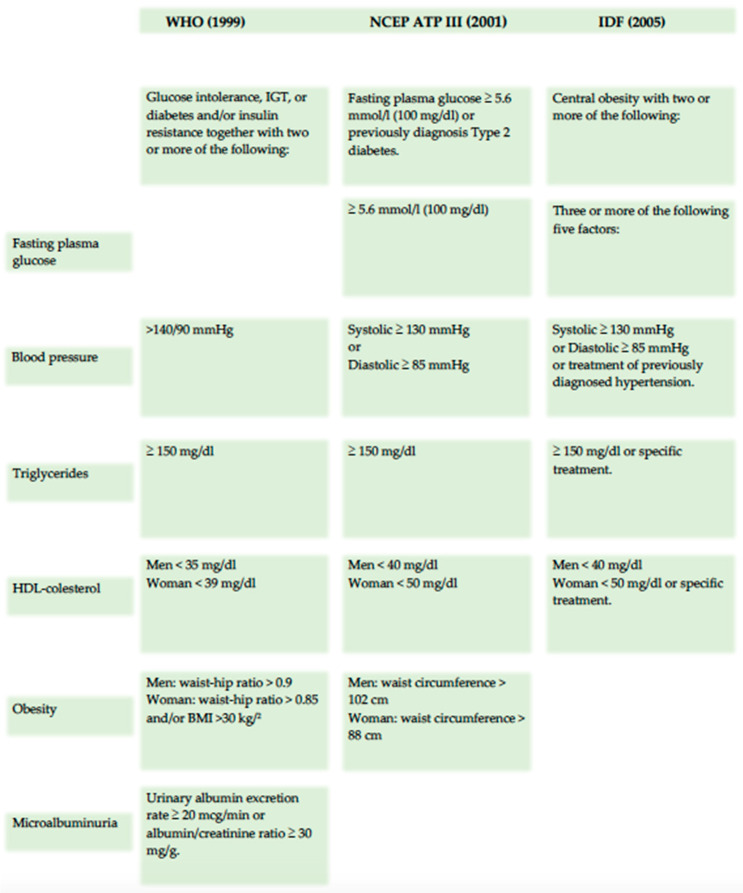
Principal metabolic syndrome definition.

The first formal definition of metabolic syndrome (MetS) was introduced by the World Health Organization (WHO) in 1998, which identified insulin resistance, along with related conditions such as impaired glucose tolerance (IGT) and type 2 diabetes mellitus, as core components of MetS. Additional features included elevated blood pressure, hypertriglyceridemia or low HDL cholesterol levels, obesity, and microalbuminuria. In contrast, the Adult Treatment Panel III (ATP III) offered a different perspective in 2001, not considering insulin resistance or central obesity as essential elements of MetS. Instead, the ATP III viewed MetS as a cluster of metabolic risk factors, placing less emphasis on its underlying pathophysiology [15]. The 2005 definition by the International Diabetes Federation (IDF) introduced central obesity as a prerequisite for diagnosing MetS, with a focus on measuring waist circumference as a simple screening tool (Figure 3).

As a result, numerous studies have evaluated the various definitions of MetS. For example, in 2006, Malek M. et al. conducted a prospective study in patients with no history of cardiovascular disease (CVD), following them for five years. The study aimed to assess the predictive value of the ATP III and IDF MetS definitions for cardiovascular disease. Different regression models were used to analyze this predictive value. Model 1 considered only MetS, while Model 2 adjusted for age, sex, family history of early CVD, and smoking. Model 3 added serum LDL levels, and Model 4 incorporated additional components of MetS. The study found that, according to both ATP III and IDF criteria, MetS was a predictor of CVD in the first three models. However, in Model 4, none of the MetS definitions predicted CVD. Notably, in Model 2, the ATP III criteria demonstrated significantly greater predictive power for CVD than the IDF criteria (AUC 0.760 vs. 0.735, *p* < 0.001). However, no significant difference was observed between the two criteria in Model 3. After adjusting for common cardiovascular risk factors and LDL, both the ATP III and IDF definitions showed similar predictive abilities for CVD. In contrast, neither definition maintained predictive power after further adjustments for their components and other factors [16]. Another prospective study published in 2007 by P. M. Nilsson et al. showed that the 2005 IDF definition of metabolic syndrome resulted in a higher prevalence rate of MetS than previous definitions (NCEP-ATPIII, EGIR) but was not superior for predicting total cardiovascular events [19]. This calls into question the usefulness of the IDF criteria if the prediction of cardiovascular risk based on the MetS is the main objective.

Although the main components of MS in different criteria are the same, some critical differences and the presence of different thresholds have caused disparities between the results of different definitions.

In 2011, Christoph H. Saely et al. [20] conducted a prospective study with 750 coronary artery patients to observe them over four years and assess the number of major cardiovascular events. Event-free survival was significantly lower (*p* < 0.001) in patients with metabolic syndrome according to the ATPIII definition than in patients who did not meet the ATPIII criteria for diagnosing metabolic syndrome. In contrast, in patients with metabolic syndrome, according to the IDF definition, event-free survival was not significantly different (*p* = 0.283) than in patients who did not meet the IDF criteria for the diagnosis of metabolic syndrome.

This result led to the conclusion that metabolic syndrome, defined by the ATPIII criteria, carries a higher risk of vascular events than metabolic syndrome, defined by the IDF criteria.

Given the challenges in establishing clear and universal criteria for MetS and the lack of definitive evidence regarding its exact pathogenesis, the Joint Interim Statement proposed a consensus definition. According to this definition, a diagnosis of MetS can be made if a patient exhibits at least three of the following criteria [21]:

Elevated waist circumference (WC), with thresholds determined by population and country-specific guidelines (≥102 cm for European men and ≥88 cm for European women) [22].

Blood triglycerides (TGs) ≥ 150 mg/dL.

Reduced blood HDL cholesterol (<40 mg/dL in men and <50 mg/dL in women).

Elevated blood pressure (BP) ≥ 130/85 mmHg.

Fasting blood glucose ≥ 100 mg/dL.

MetS has become a significant public health concern due to its high prevalence. The medical and scientific communities have emphasized the urgency of defining strategies to address this growing pandemic. The prevalence of MetS varies globally. For instance, Aguilar et al. [23] reported an overall prevalence of 33% in the United States in 2012, while Liang et al. [24] noted an increase to 38.3% in 2018. In 2017, the prevalence reached 48.8% in Qatar [25] and 42.87% in Iran [26].

As a preventable condition, MetS is closely linked to unbalanced dietary habits and a Westernized lifestyle, patterns often observed from childhood. Preventative measures such as adopting a healthy diet and engaging in regular physical activity have shown protective effects against MetS. Notably, studies highlight the Mediterranean diet’s role in significantly reducing the risk of metabolic syndrome [27,28,29].

### 3.3. Future Direction of Cardiovascular and Metabolic Diseases

In a 2024 editorial published in *Biomedicines* titled “Cardiovascular and Metabolic Disease: New Treatments and Future Directions” (A. Caturano), the authors emphasize that, in recent decades, cardiovascular disease (CVD) and metabolic disorders have become significant global health challenges, driving up healthcare costs. Despite advancements in medical science and technology, these conditions remain leading contributors to morbidity and mortality, highlighting the critical need for innovative approaches in prevention, diagnosis, and treatment [27,28].

A substantial proportion of CVD-related deaths are closely linked to the presence of metabolic disorders, particularly the components of metabolic syndrome (MetS). Addressing these interconnected risks requires thorough understanding and targeted interventions [29,30].

Behavioral risk factors such as tobacco use, poor dietary habits, physical inactivity, and excessive alcohol consumption exacerbate the vulnerability to both cardiovascular disease and metabolic disorders. Thus, tackling these modifiable risk factors is essential in any comprehensive strategy aimed at disease prevention and management [30,31,32]. In light of this, the role of the Mediterranean diet, not only as a healthy lifestyle but also as a significant player in preventing metabolic diseases related to cardiovascular risk, fits perfectly.

Looking to the future, the research landscape on cardiovascular and metabolic diseases promises transformative advances and innovative interventions.

Personalized medicine is increasingly recognized as a key approach in the management of cardiovascular and metabolic diseases. The advent of precision medicine and genomic technologies has opened up new avenues for customizing interventions based on the distinct characteristics and needs of individual patients. This strategy improves therapeutic outcomes while minimizing adverse effects. By utilizing advanced data analysis and artificial intelligence, researchers can explore the intricate interactions between genetic, environmental, and lifestyle factors that affect disease susceptibility and progression, thereby enabling the development of more targeted and effective treatments [33].

Metabolic syndrome (MetS) encompasses a cluster of risk factors for cardiovascular disease (CVD), making the treatment and management of its components a crucial goal for preventing chronic metabolic conditions and reducing CVD-related mortality. Traditionally, MetS diagnosis involves simple measurements such as waist circumference (WC), blood pressure, HDL cholesterol (HDL-C), triglycerides, and blood glucose levels. However, metabolomic techniques, such as NMR spectroscopy and chromatographic methods, can identify a broader range of metabolites linked to MetS and its individual components. These molecular biomarkers could become valuable tools for diagnosing and predicting MetS in affected individuals.

In addition, various nutrients, bioactive food compounds, and dietary patterns have been explored for their potential therapeutic effects in managing MetS. Several nutraceuticals show promise as dietary supplements due to their accessibility and beneficial properties (see Table 2 and Figure 3). While a number of nutrients (see Table 2 and Figure 3) have demonstrated potential in addressing specific MetS components, no single dietary strategy has been extensively studied for the comprehensive management of MetS. Recently, there has been increasing interest in the role of gut microbiota and its modulation through prebiotics and probiotics, which could provide valuable insights into the pathophysiology of MetS and lead to innovative management approaches for the metabolic disturbances that underlie the syndrome. Furthermore, some studies suggest that polyphenol intake may offer protective and beneficial effects against MetS [34]. However, further research is needed to deepen our understanding of the health impacts of polyphenols. Such findings could pave the way for the development of targeted polyphenol supplementation strategies to maximize their therapeutic potential.

The future of research in cardiovascular and metabolic diseases holds immense promise for innovation and transformation. By embracing a multidisciplinary approach, integrating cutting-edge technologies, and focusing on patient-centered care, we can work towards a future where the burden of these diseases is significantly reduced, improving health and well-being for individuals and communities worldwide.

## 4. Cardiometabolic Risk: The Scores

In the decade between 1956 and 1966, investigators in Framingham, Mass, defined age, hypertension, smoking, diabetes, and hyperlipidemia as significant determinants of coronary heart disease and coined the term coronary risk factors [33,34,35,36]. Over time, these markers were codified into global risk scores for the assessment of cardiovascular risk [37,38].

Several widely used and scientifically approved cardiovascular risk scores arose from the need to develop a patient’s risk profile to prevent major cardiovascular events or intervene with increasingly personalized therapies.

Among these, the most widely used are the following:

Framingham risk score: Based on the findings of the Framingham studies, this score calculates the ten-year risk of developing cardiovascular disease (CVD) for men and women. It incorporates factors such as age, sex, cholesterol levels, blood pressure, smoking habits, and family history. However, this method may fail to identify individuals who have a low short-term but high lifetime risk of coronary heart disease, likely due to variations in risk factor status over time [39].

ASCVD Risk Calculator: Developed by the American College of Cardiology (ACC) and the American Heart Association (AHA), this tool estimates the 10-year risk of atherosclerotic cardiovascular events (ASCVDs). It utilizes factors similar to those in the Framingham score but places particular emphasis on atherosclerotic diseases [40].

SCORE (Systematic Risk Evaluation): Primarily utilized in Europe, the SCORE system evaluates the risk of cardiovascular mortality based on factors such as age, sex, blood pressure, cholesterol levels, and smoking habits [40].

SCORE 2 (Systematic Coronary Risk Evaluation 2): A newly developed, calibrated, and validated algorithm for predicting the 10-year risk of first-onset CVD in European populations improves the ability to identify individuals at higher risk of developing CVD across Europe [41].

SCORE 2-OP (Old Person): The competing risk-adjusted SCORE2-OP model was developed, recalibrated, and externally validated to estimate 5- and 10-year CVD risk in adults aged 70 years or older across four geographical risk regions. These models help convey the risk of CVD and the potential benefits of risk factor management, supporting shared decision making between clinicians and patients in managing CVD risk in older populations [42].

QRISK-SCORE: The first QRISK model, designed to estimate the 10-year risk of cardiovascular disease, was introduced in 2007. It was followed by an updated version, QRISK2, in 2008, which incorporated ethnicity and additional risk factors such as type 2 diabetes, rheumatoid arthritis, atrial fibrillation, and chronic kidney disease. Since then, QRISK2 has been updated annually, extending the age range to 25–84 years, including type 1 diabetes as a separate variable, and refining smoking into five categories. Recent studies have emphasized an increased cardiovascular risk and the potential prognostic significance of erectile dysfunction [43], migraine [44], and blood pressure variability [45]. QRISK3 was developed to evaluate whether incorporating these factors into the algorithm could enhance cardiovascular risk estimation for affected patients [46].

The Reynolds Risk Score: In women, up to 20% of all coronary events occur without the presence of major risk factors [47], while many women with traditional risk factors do not experience coronary events [48]. Additionally, over the past 50 years, the understanding of the biological mechanisms driving atherothrombosis has expanded significantly to include the intricate roles of hemostasis, thrombosis, inflammation, endothelial dysfunction, and plaque instability [49,50]. Researchers have developed two clinical algorithms for global cardiovascular risk prediction, which reclassified 40% to 50% of intermediate-risk women into higher- or lower-risk categories [51].

## 5. Effects of Mediterranean Diet on Cardiovascular Risk Factors and Metabolic Syndrome

Metabolic syndrome risk factors can be categorized into two types: modifiable and non-modifiable. Modifiable factors such as diet, physical activity, and smoking present opportunities for individuals to take action and lower their risk of metabolic syndrome [52]. Among dietary patterns, the Western diet is particularly associated with a higher likelihood of developing metabolic syndrome [53]. This is due to its composition, which includes substantial amounts of red meat, processed meat products, refined cereals, high-fat, low-fruit dairy products, vegetables, nuts, and legumes [54]. Key components of the Western diet—saturated fats, trans fats, omega-6 (*n*-6) fatty acids, simple carbohydrates, sucrose, and salt—are abundant, while it lacks complex carbohydrates, dietary fiber, and omega-3 fatty acids [39]. These dietary imbalances contribute to oxidative stress, inflammation, dyslipidemia, and non-communicable diseases like obesity and its associated complications [40].

In addition to dietary habits, a sedentary lifestyle is another modifiable risk factor for metabolic syndrome [41]. Regular physical activity can counteract these risks by increasing energy expenditure, enhancing lipolysis, improving insulin sensitivity, lowering blood pressure, and optimizing blood lipid parameters [42]. Consequently, a sedentary lifestyle is closely linked to an elevated risk of metabolic syndrome.

**Figure 4:**
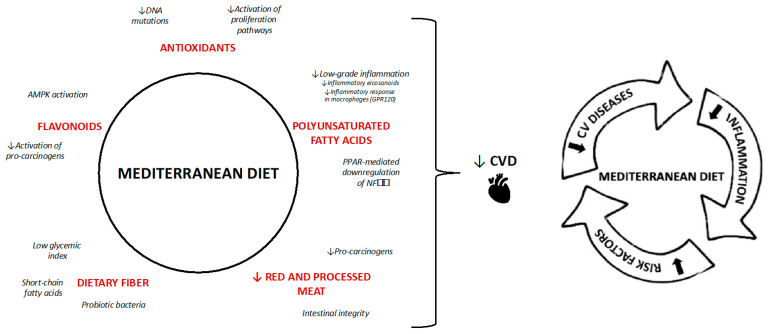
Mechanisms of Mediterranean diet. The black arrows means lower incidence of CVS and the red arrows indicate “lower”.

Smoking, widely regarded as one of the most detrimental habits, exacerbates metabolic syndrome risk by contributing to abdominal fat accumulation, visceral obesity, insulin resistance, dyslipidemia, hypertension, and other health issues—all of which are linked to oxidative stress and inflammation [43]. Similarly, excessive alcohol consumption raises health risks, including visceral obesity, reduced insulin sensitivity, hypertension, and abnormal lipid profiles [44]. The Mediterranean diet is the most effective nutritional model, potentially reducing chronic non-communicable disorders thanks to its contents. It includes numerous beneficial nutrients that can negatively affect metabolic pathways altered by chronic diseases and, of course, potentially impact the metabolic syndrome (Figure 4).

Olive oil, a fundamental element of the Mediterranean diet, contains polyphenols that are instrumental in mitigating the risk of metabolic syndrome by addressing factors such as visceral obesity, insulin resistance, hypertension, and lipid peroxidation. These polyphenols suppress the activation and expression of nuclear factor kappa B (NFKB), a pivotal component in metabolic syndrome, thereby lowering the secretion of proinflammatory cytokines [55,56]. Another defining feature of the Mediterranean diet is red wine, whose main polyphenol, resveratrol, possesses notable anti-inflammatory and antioxidant properties [57,58]. Resveratrol is also associated with modulating the human gut microbiota, a key element in metabolic health, activating sirtuin 1 essential for lipolysis, and stimulating adenosine monophosphate-activated protein kinase, which enhances insulin sensitivity.

Citrus fruits, prevalent in the Mediterranean region, contain polyphenols that help reduce advanced glycation end products and suppress NFKB expression, thereby diminishing oxidative stress and inflammation in the body. This reduction improves insulin sensitivity, lipid metabolism, and blood pressure. Furthermore, polyphenols such as naringenin promote energy metabolism, aiding in the reduction in visceral fat [59]. Mediterranean vegetables, fruits, and spices are also abundant in polyphenols, which are bioactive compounds capable of inhibiting oxidative stress and inflammation-related pathways. These mechanisms elevate plasma HDL levels, reduce LDL levels, and improve insulin resistance, body mass index, and blood pressure [60]. Additionally, dietary polyphenols act as effective prebiotics, curbing harmful microorganisms and fostering beneficial gut microbiota. A balanced gut microbiota correlates with better glucose tolerance, enhanced insulin secretion, reduced lipogenesis, and decreased inflammation [61]. Ultimately, the polyphenols characteristic of the Mediterranean diet alleviate inflammation, oxidative stress, insulin resistance, lipid oxidation, body weight issues, blood pressure, and endothelial dysfunction, thereby addressing key risk factors associated with metabolic syndrome [62].

The Mediterranean diet is also rich in beneficial fatty acids, particularly *n*-9 and *n*-3 fatty acids, due to frequent olive oil consumption and moderate fish intake while being low in saturated and trans fats. Omega-9 fatty acids, especially oleic acid, exhibit antioxidant and anti-inflammatory effects that enhance pancreatic beta-cell function, insulin sensitivity, and endothelial function [63]. Oleic acid also modulates hypothalamic function, lowers ghrelin secretion, and inhibits platelet aggregation. Moreover, it reduces plasma LDL levels while increasing HDL concentrations [64]. In addition, the combined effects of oleic acid and polyphenols in olive oil regulate blood pressure by inhibiting the ACE pathway [65]. Omega-3 fatty acids further contribute to addressing metabolic syndrome criteria [66,67].

Dietary fiber, both soluble and insoluble, also provides notable benefits for managing metabolic syndrome [68]. Soluble fiber increases intraluminal viscosity, slowing gastric emptying and macronutrient absorption, thereby assisting in weight management and controlling postprandial blood glucose levels. During fermentation, it produces short-chain fatty acids that inhibit glucose production and fatty acid synthesis in the liver while reducing macronutrient absorption via enterocytes [69]. Additionally, soluble fiber reduces bile acid reabsorption, compelling the liver to synthesize more bile acids using cholesterol, which subsequently lowers plasma cholesterol levels [70].

Conversely, insoluble fiber enhances chewing time and reduces colon transit time, activating the vagus nerve and promoting satiety. These effects lower food intake and nutrient absorption, supporting the reduction in obesity and insulin resistance.

Vitamins A, C, and E play a crucial role in defending against oxidative stress, a key contributor to various non-communicable diseases such as insulin resistance, cardiovascular disease, and cancer [71]. These antioxidants alleviate stress on pancreatic beta cells and tissues, improving insulin sensitivity and secretion, critical for combating insulin resistance and supporting weight management [72]. Additionally, they help reduce proinflammatory cytokines and reactive oxygen species, enhancing endothelial function and benefiting blood pressure regulation, lipid metabolism, and overall cardiovascular health [73], see Figure 4.

Lastly, the Mediterranean diet is characterized by a lower intake of harmful substances, including saturated and trans fats, linoleic acid (*n*-6), cholesterol, simple carbohydrates, sodium, nitrites, and nitrates, alongside reduced total fat consumption. Given these advantages, the Mediterranean diet represents a promising strategy for reducing the risk of metabolic syndrome and promoting longevity.

### 5.1. Mediterranean Diet and Diabetes

Although type 2 diabetes (T2D) is a multifactorial condition influenced by both genetic and environmental factors, healthy lifestyle choices, including an appropriate diet, are considered crucial in its development [74]. Therefore, we will focus on the potential role of the Mediterranean diet in mechanisms related to T2D. As previously mentioned, numerous studies have demonstrated a beneficial impact of the Mediterranean diet on T2D.

The Mediterranean diet can help reduce central obesity, which in turn lowers the risk of obesity-related chronic diseases, such as T2D [75]. It has also been shown to be more effective than a low-fat diet for long-term weight loss [76]. Thus, it is the composition of the Mediterranean diet, rather than its caloric content, that likely contributes to its positive effects on T2D. Furthermore, although the individual effects of specific dietary components may be too subtle to detect on their own, their combined influence could be substantial enough to be noticeable [77].

The role of the Mediterranean diet in preventing type 2 diabetes (T2D) has been emphasized in a review conducted by Sandra Martín-Peláez and colleagues [45].

Recent studies demonstrated a significant association between adherence to dietary patterns and a reduced risk of T2D [46,47,48]. For instance, a 2014 systematic review and meta-analysis by Schwingshackl et al. [49] revealed that greater adherence to a Mediterranean diet was linked to a 19% reduction in diabetes risk (moderate quality evidence). This research suggested that the protective effects of the Mediterranean diet against diabetes were more pronounced in European populations than in North American ones and highlighted the long-term benefits of dietary adherence in studies lasting over a decade. These findings underline the importance of promoting Mediterranean-style dietary patterns as a public health strategy for the primary prevention of T2D.

Data from the PREDIMED trial, a large multicenter randomized controlled study, showed that a Mediterranean diet enriched with extra virgin olive oil (EVOO) or nuts significantly reduced diabetes risk by 52% in elderly individuals with high cardiovascular risk compared to a low-fat diet [48]. This protective effect was attributed to the overall dietary composition rather than caloric restriction, increased physical activity, or weight loss.

In 2017, a systematic review and meta-analysis of nearly 1.5 million participants compared various dietary models and confirmed the Mediterranean diet, alongside other heart-healthy diets, as effective in reducing diabetes risk [45,46,47,78,79]. Among these was the DASH (Dietary Approaches to Stop Hypertension) diet [49], which focuses on managing sodium intake (1500 to 2300 mg/day) and encouraging the consumption of low-fat vegetables, fruits, dairy products, whole grains, fish, poultry, and nuts. Another included diet was the AHEI (Alternative Healthy Eating Index), which evaluates adherence to American dietary guidelines. Both DASH and Mediterranean diets demonstrated a 20% reduction in T2D risk, with no significant differences in outcomes based on follow-up duration or geographical context [80]. These diets shared key components, such as olive oil, whole grains, fruits, vegetables, legumes, nuts, moderate alcohol consumption, and low intake of red/processed meats and sugary beverages [80].

Vegetarian diets, which emphasize whole plant foods, offer various eating patterns that exclude or limit animal products. Veganism, the most restrictive form, avoids all animal products, while lacto-ovo-vegetarians include dairy and eggs but avoid meat. Fish-vegetarians add fish to their diet, and semi-vegetarians consume limited amounts of meat and meat products. Studies indicate an inverse association between vegetarian diets and T2D risk, especially given the established link between meat consumption and diabetes development. For example, the 2018 Rotterdam study found that a vegetarian diet reduced diabetes risk by 18% and prediabetes risk by 11% over a four-to-seven-year follow-up period [81].

Regarding the Mediterranean diet, the ATTICA study revealed that adherence to this dietary pattern improved fasting glucose homeostasis, insulin levels, and insulin resistance (the HOMA index) in individuals with and without diabetes [82]. Participants with higher Mediterranean diet adherence scores experienced a 15% reduction in basal glucose and insulin levels and a 27% improvement in the HOMA index [82]. Furthermore, the PREDIMED study involving 722 high-cardiovascular-risk participants reported that those on a Mediterranean diet enriched with olive oil or nuts showed decreases in fasting blood glucose of 0.39 and 0.30 mmol/L, respectively, compared to the control group, without weight loss over three months [83].

The impact of specific Mediterranean diet components, such as olive oil, has also been extensively studied. A randomized crossover study found that moderate daily consumption (25 mL/day) of virgin olive oil containing 577 mg/kg phenolic compounds over eight weeks reduced fasting blood glucose and HbA1c levels in overweight individuals with T2D [84]. Additionally, a cohort study of 4903 Italian adults aged 20–59 years established a correlation between monounsaturated fatty acids, the main component of olive oil, and lower plasma glucose levels [85,86]. A cross-sectional study in Spain (PIZARRA) further demonstrated that individuals consuming olive oil had lower insulin resistance compared to those using sunflower oil or mixed oils [87].

### 5.2. Mediterranean Diet and Hypertension

The 2024 Guidelines define hypertension as a confirmed office systolic blood pressure (BP) of ≥140 mmHg or diastolic BP of ≥90 mmHg. To establish this diagnosis, it is recommended to confirm the measurement with out-of-office assessments (such as HBPM or ABPM) or at least one repeat office measurement during a follow-up visit. The risk of adverse cardiovascular disease (CVD) outcomes rises in a log-linear fashion with consistent increases in both systolic and diastolic BP [88,89,90,91,92]. Additionally, at higher BP levels, other CVD risk factors tend to cluster [93,94]. As a result, many individuals with hypertension will have an estimated 10-year risk of CVD events ≥10% [50,51,95,96,97,98,99,100], which is considered sufficiently high to warrant BP-lowering treatment in cases of elevated BP according to these guidelines.

Following the ESC 2024 guidelines, lifestyle interventions are prioritized to improve treatment effectiveness and prevent hypertension.

The benefits of a healthy lifestyle are now known in a range of results, extending well beyond the blood pressure-lowering effects [50], but also include positive effects on mental and physical health, which are considered a Class I recommendation.

### 5.3. Mediterranean Diet and Lipid Levels

Metabolic syndrome (MetS) is associated with lipoprotein structure and function alterations that can be characterized by advanced lipoprotein testing (ADLT). Atherosclerosis represents the primary cause of cardiovascular diseases [51], which is because cholesterol-rich lipoproteins and apolipoprotein B become oxidized in the arterial subendothelial matrix, and this process determines the production of proinflammatory molecules, which activates the endothelium itself. This process is also mediated by monocytes recruited from the bloodstream. Once reaching the site, these cells become foamy after phagocytizing the oxidized LDL, contributing to the increase in the plaque’s size and the eroticization of the inflammatory process [100,101].

In a study published in 2024 by B. Candás-Estébanez et al., the effects of the MedDiet alone and an energy-reduced MedDiet intervention in addition to physical activity were compared) (er-MedDiet + PA) on the profiles of lipoprotein subclasses in patients with metabolic syndrome (MetS). The single MedDiet improved lipoprotein composition with a reduction in HDL triglyceride (HDL-TG) and small dense-LDL cholesterol (sd-LDL-C) and an increase in ILDL particles, while the er-MedDiet + PA intervention led to an improvement in triglyceride metabolism (TG). It is important to note that these changes in TG metabolism are associated with an improvement in the composition of LDL. The combination of the MedDiet with negative energy balance obtained through diet and physical activity has proven to improve TG-rich lipoprotein metabolism and reduce atherogenic lipoprotein concentration. The results illustrate that, without lifestyle changes, lipid-lowering treatment alone cannot reduce cardiovascular risk in patients with metabolic syndrome (MetS) [102].

The therapeutic strategies for treating these conditions range from medical interventions to lifestyle modifications [101]. In this context, various substances found in Mediterranean diet products have been studied for their potential cardiovascular benefits. Extra virgin olive oil, omega-6 and omega-3 fatty acids from nuts, and plant sterols are key sources of fatty acids in the Mediterranean diet [67].

The increased intake of water-soluble fibers, such as those found in fruits and beans, has been shown in numerous randomized controlled studies to significantly reduce plasma LDL cholesterol levels (each gram of fiber results in a reduction of approximately 1.12 mg/L of LDL cholesterol) [102,103]. Another beneficial effect is the reduced reabsorption of cholesterol and bile acids in the intestine, which then promotes their absorption in the liver. Extra virgin olive oil contains significant amounts of tocopherol, carotenoids, oleuropein, and phytosterols in every 100 g. Among these, phytosterols, such as ibuprofen, appear to inhibit COX activity [104]. The amount of phytosterols in 50 g of extra virgin olive oil may not have strong anti-inflammatory effects but is sufficient to offer protection against platelet aggregation.

Carotenoids, including α-carotene, β-carotene, lycopene, lutein, fucoxanthin, canthaxanthin, zeaxanthin, β-cryptoxanthin, capsorubin, and astaxanthin, are primarily found in fruits and vegetables. They possess antioxidant properties and play a role in regulating the immune system and cell signal transduction [105]. Carotenoids are also involved in the atherosclerotic process, helping to slow the progression of atherosclerotic plaque [106,107]. A 2023 study conducted by G. Del Castillo Vidal et al. demonstrated that the consumption of tomatoes (rich in carotenoids), both alone and in combination with extra virgin olive oil, contributed to a reduction in plasma cholesterol levels.

Nuts, fruits, and vegetables also contain another antioxidant: coenzyme Q [108,109].

### 5.4. Mediterranean Diet and Obesity

Visceral obesity is commonly associated with arterial hypertension [110]. An average weight loss of 5 kg was associated with an average systolic and diastolic pressure reduction of 4.4 and 3.6 mmHg, respectively [111]. Data show that, starting from a body mass index (BMI) of 40 kg/m^2^, an average weight loss of 13% is associated. The risk of hypertension is lowered by 22% [112,113]. To understand the molecular mechanisms behind visceral fat-related diseases, several studies have examined the biological characteristics of both visceral and subcutaneous adipose tissue by analyzing their gene expression profiles. Among the genes categorized by function and location, approximately 20% of all genes in subcutaneous adipose tissue encode secretory proteins, while around 30% do so in visceral adipose tissue. These bioactive substances are known as ‘adipocytokines’ and are further divided into two categories: adipose tissue-specific bioactive substances (e.g., leptin and adiponectin) and those that are abundantly secreted by adipose tissue but are not specific to it (e.g., PAI-1, tumor necrosis factor α, interleukins, and others). Adipocytokines play a crucial role in regulating glucose and lipid metabolism, controlling oxidative stress, and maintaining the integrity of the vascular wall. For instance, TNF-α, IL-6, and leptin can induce insulin resistance, while adiponectin enhances insulin sensitivity. Several studies indicate that leptin may serve as a key connection between central obesity, hypertension, and metabolic syndrome (MetS) [114].

A weight loss of 5–10% of initial body weight can improve the cardiovascular risk profile as a control of BP, maintain adequate glucose and lipid metabolism, and potentially reduce premature mortality from all causes [55,56,115].

Weight stabilization during middle age seems an essential and achievable goal in preventing obesity-related blood pressure rise later in life [57]. Evidence-based diets, such as the Mediterranean diet and Dietary Approaches to Stop Hypertension (DASH) are well-established interventions to reduce blood pressure and the risk of cardiovascular disease [2]. In the CARDIOPREV study, J. Delgrado-Lista et al. showed that the Mediterranean diet was superior to the low-fat diet in preventing major cardiovascular events [58]. In recent years, more and more studies have shown the efficacy and safety of weight reduction and cardiovascular risk of peptide one receptor agonists (GLP-1) [60,116]. For example, in the STEP-1 study, semaglutide treatment combined with a proper lifestyle in obese people resulted in an average weight reduction of 124%, a 5.1 mmHg reduction in systolic pressure and an improvement in cardiovascular risk [61].

## 6. Conclusions

The effects of the Mediterranean diet on the components of metabolic syndrome highlight its protective role against cardiometabolic risk. Since the 1960s, evidence gathered from clinical and epidemiological studies supports the hypothesis that adherence to this dietary pattern, characterized by the high consumption of fruits, vegetables, whole grains, fish, and olive oil, contributes to improving various metabolic parameters, such as central obesity, blood glucose levels, and lipid profiles. Over time, modern studies have increasingly confirmed the hypotheses regarding the mechanisms underlying the benefits of the Mediterranean diet. The components of the Mediterranean diet, enriched with bioactive compounds, are associated with a reduction in risk factors for cardiovascular diseases, including lipoprotein abnormalities, obesity, metabolic syndrome, diabetes mellitus, and hypertension. Supplementing this diet with olive oil or nuts further mitigates cardiovascular risk factors and suppresses cellular and humoral inflammatory pathways linked to atherosclerosis, potentially enhancing endothelial function.

Research indicates that the Mediterranean diet lowers the incidence of metabolic syndrome, improves insulin sensitivity, reduces systemic inflammation, and significantly decreases cardiovascular risk. Its widespread applicability across various populations underscores its value as a versatile and effective therapeutic approach for managing cardiometabolic risks. In the context of modern medicine, which prioritizes prevention, adopting a healthy and active lifestyle alongside adherence to the Mediterranean diet—centered on simple, wholesome foods—holds immense promise and can contribute to an increased life expectancy for the population and a reduction in public healthcare costs. Thus, in a modern society that has indulged us with comforts, the key to longevity may be to return to the simple lifestyle of the past, guided by the advancements of medical sciences.

## Figures and Tables

**Table 1:** List of foods of the typical Mediterranean diet.

Foods of the Mediterranean Diet
vegetables
fruits
extra virgin olive oil
wholegrain breads and cereals
legumes or beans
low-fat dairy
poultry
fish
nuts and seeds
fish and seafood
onion, garlic and other herbs and spices (e.g., oregano, coriander, cumin, etc.)

**Table 2:** List of Mediterranean diet major bioactives/nutrients.

Mediterranean-Style Major Bioactives/Nutrients
Dietary fibers
Increased ratio of MUFA to SFA
Antioxidants
Zn
Iodine
Mg
Fe
Calcium
Vitamin B12
Vitamin B6
Vitamin B3
Vitamin B2
Vitamin B1
Vitamin A
Vitamin C
Vitamin D
Vitamin E
Vitamin A
Se
Folic acid

Zn: zinc; Mg: magnesium; Se: selenium; MUFA: monounsaturated fatty acid; and SFA: saturated fatty acid.
